# Genomic and non-genomic actions of glucocorticoids in asthma

**DOI:** 10.4103/1817-1737.65040

**Published:** 2010

**Authors:** Abdullah A. Alangari

**Affiliations:** *Department of Pediatrics, College of Medicine, King Saud University and King Khalid University Hospital, Riyadh, Saudi Arabia*

**Keywords:** Asthma, genomic action, glucocorticoids, mechanism of action, non-genomic action

## Abstract

Glucocorticoids are the mainstay of asthma therapy. They are primarily used to suppress airway inflammation, which is the central pathological change in asthmatic patients’ airways. This is achieved by many different mechanisms. The classical mechanism is by suppression of the genetic transcription of many inflammatory cytokines that are key in asthma pathophysiology (transrepression). On the other hand, the transcription of certain inhibitory cytokines is activated by glucocorticoids (transactivation), a mechanism that also mediates many of the adverse effects of glucocorticoids. The onset of action through these mechanisms is often delayed (4-24 hours). Other mechanisms mediated through non-genomic pathways are increasingly appreciated. These are delivered in part by binding of glucocorticoids to nonclassical membrane-bound glucocorticoid receptors or by potentiating the α1-adrenergic action on the bronchial arterial smooth muscles, in addition to other mechanisms. These effects are characterized by their rapid onset and short duration of action. Understanding these different mechanisms will help in the development of new and better drugs to treat this common disease and to develop new improved strategies in our approach to its management. Here, the genomic and non-genomic mechanisms of actions of glucocorticoids in asthma are briefly reviewed, with special emphasis on the current updates of the non-genomic mechanisms.

Asthma is one of the most common chronic illnesses worldwide that has a huge burden on every society. It is the most common chronic illness in childhood and is one of the most common causes of admission to the hospital among children and adults.[1,2] Its prevalence varies from 5% to 30% based on the population studied. In developed countries the prevalence increased during the 70s and 80s and now has reached a plateau. However, it is still rising in many developing countries.[3–5] In Saudi Arabia, for instance, the prevalence of asthma in children was found to be 15% to 23% based on the studied area.[6] In another study as well in Saudi Arabia, it was found that about 5.7% of all emergency room (ER) visits were because of asthma exacerbations.[7]

Recent data from the United States indicate improved outcomes of asthma morbidity and mortality, with fewer annual hospitalizations for asthmatic attacks and fewer asthma-related deaths.[3] This could partly be attributed to the more widespread preventive use of inhaled glucocorticoids, the gold standard asthma therapy.[8] Glucocorticoids have been used in the treatment of asthma for over half a century, and inhaled glucocorticoids were first introduced in 1972. Since that time vast experience has been gained and a huge body of literature has been developed in this area.[9] Systemic glucocorticoids are mainly used to treat acute asthma exacerbations. On the other hand, inhaled glucocorticoids either in dry powder form or as metered-dose inhaler (MDI) are used as daily-maintenance therapy to control asthma symptoms and prevent exacerbations. Currently, inhaled glucocorticoids are the best available medications that alleviate asthma symptoms, improve baseline pulmonary function, reduce airway hyper-responsiveness and decrease asthma morbidity and mortality.[8–11]

In this short review I will discuss, focusing on recent updates, the different genomic and non-genomic mechanisms by which glucocorticoids act.

## Genomic Action

The *genomic action* of glucocorticoids implicates the activation or repression of multiple genes. Glucocorticoids significantly suppress airway inflammation, mainly through genomic mechanisms. This action involves many steps and therefore takes effect with a time lag of about 4 to 24 hours.[12] So far all the recommended uses of glucocorticoids in asthma therapy are related to this mode of action, including the use of systemic glucocorticoids in patients with asthma exacerbation in the ER.[13,14]

**Figure 1:**
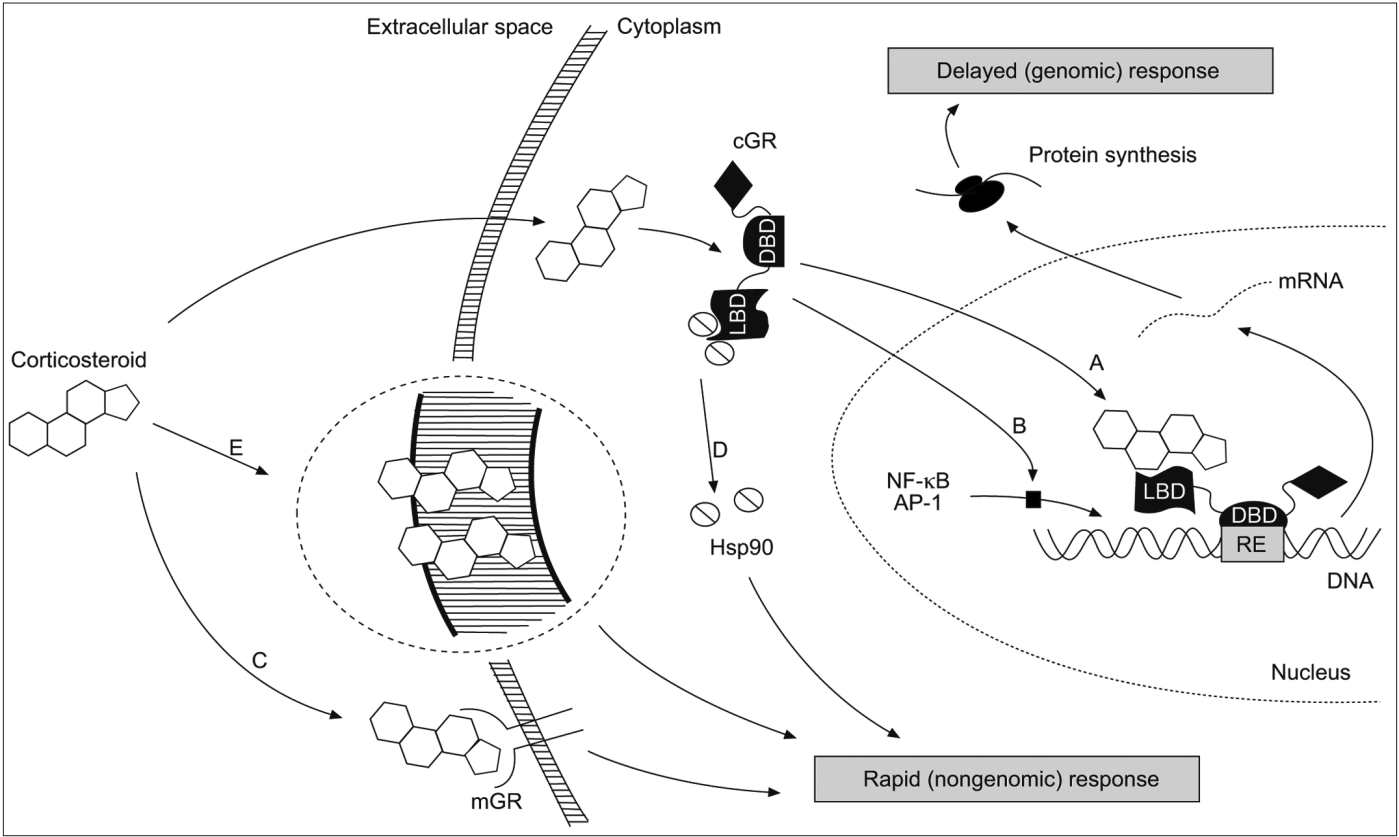
Genomic actions are mediated through a) direct DNA binding (transactivation) or b) transcription factor inactivation (transrepression). Non-genomic actions are mediated by c) membrane-bound receptors, d) cytosolic receptors or e) interaction with cell membrane. cGR: cytosolic glucocorticoid receptor; mGR: membrane-bound glucocorticoid receptor; LBD: ligand-binding domain; DBD: DNA-binding domain; hsp 90: heat-shock protein 90; RE: response element; NFkB : nuclear factor kB; AP-1: activating protein-1. (Adopted from: Horvath, G and Wanner, A. Eur Respir J. 2006;27:172-187 with permission)

Glucocorticoids are lipid-soluble molecules that can freely pass through the cell membrane. Their genomic actions are mediated through binding to their ubiquitously expressed cytoplasmic glucocorticoid receptors (GRs). The GR gene is located on chromosome 5q31-q32 and is composed of 9 exons. GR has 2 main isoforms, α and β, which are produced by alternative splicing of exon 9.[15] Isoform α is ubiquitously expressed and mediates the genomic actions of glucocorticoids. On the other hand, isoform β accounts for 0.2% to 1% of total GR expression and is unable to bind glucocorticoids.[16] It may contribute to glucocorticoids’ resistance through the formation of GRα/GRβ heterodimers.[17] GRs are members of the steroid hormone receptor super-family, which includes receptors for mineralocorticoids, estrogens and androgens, in addition to vitamin D_3_ and thyroid hormones. They share a common structure, which contains ligand-binding domain, DNA-binding domain and an activity-modulator domain.[16] The GR is present in the cytoplasm in an inactive state as a multimeric complex with different proteins like heat shock protein 90 (hsp90), hsp70, hsp56, hsp40; and immunophilins like p23 and src.[18] When GR binds its ligand, these proteins dissociate and the receptor becomes active [Figure 1]. Upon activation, GRs translocate to the nucleus, dimerize and bind directly to specific sites on the DNA called glucocorticoids’ response elements (GREs) or to different transcription factors (protein-protein interaction) as monomers.[19] It is estimated that there are 10 to 100 genes per cell that are directly regulated by glucocorticoids.[17,20]

## Gene Activity Modulation by Direct DNA Binding

Binding of GR to its GRE can cause gene activation, leading to increased transcription of some anti-inflammatory genes such as annexin 1, interleukin 10 (IL-10) and inhibitor of nuclear factor kappa B (ikB), a process called *transactivation* [Figure 1].[21,22] Less importantly, it can repress gene activation by binding to negative GRE, causing gene silencing by competing with other transcription factors or displacing them from the DNA. Genes affected by this mechanism include prolactin and osteocalcin.[23] However, these mechanisms are thought to have minor role in the anti-inflammatory effect of glucocorticoids. On the contrary, transactivation mechanisms probably contribute to many of the adverse effects of glucocorticoids by enhancing the expression of genes involved in different metabolic processes, leading to clinical manifestations like diabetes and glaucoma.[24,25]

## Gene Activity Modulation by Protein-protein Interaction

GR can regulate gene activity indirectly by binding to transcription factors that up-regulate or down-regulate gene transcription. For example, it can activate certain genes’ expression by binding to several STAT (signal transducer and activator of transcription) proteins, leading to a wide range of immunomodulatory actions.[26] More relevant to asthma airway inflammation, GR can repress the activity of many important pro-inflammatory genes by binding to and inhibiting key transcription factors, like nuclear factor kappa B (NFkB) and activator protein 1 (AP-1), among others, a process called *transrepression* [Figure 1].[27] Classical gene targets of NFkB that can be inhibited by its binding to GR include tumor necrosis factor α (TNFα), IL-1β and granulocyte-monocyte colony–stimulating factor (GMCSF). These cytokines play a major role in the pathophysiology of airway inflammation in asthma, including vasodilation, increased vascular permeability and inflammatory cell recruitment.[28] Targeted drug therapies for many of these cytokines are under clinical trials, and some have already shown promise in the treatment of severe asthma.[29] In addition, NFkB can induce the expression of nitric oxide synthase (NOS), which stimulates the production of nitric oxide (NO).[30] NO contributes to many of the inflammatory manifestations of asthma, including vasodilation and inflammatory cell recruitment. By binding NFkB, glucocorticoids can suppress the production of NO through inhibition of NOS gene expression.[31,32] Gene targets for AP-1 include collagenase, stromelysin and other metalloproteinases.[23] These cytokines are important players in the process of remodeling observed in asthmatic patients’ airways.[28,33] Other transcription factors that can be inhibited in the same way include smad3, which is downstream from transforming growth factor β (TGFβ) receptor.[34] Activation of this pathway is known to be important to cellular differentiation of fibroblasts to myofibroblasts, extracellular matrix deposition and impairment of epithelial repair — all are important for the process of remodeling in asthma.[35]

The development of glucocorticoids has passed through different stages since the introduction of cortisone, which has glucocorticoid and mineralocorticoid activity, to the development of prednisone, which has less mineralocorticoid activity, and then the development of glucocorticoids with almost no mineralocorticoid activity, like dexamethasone.[36] Advanced understanding of the above mechanisms and knowledge of the detailed tertiary structure of glucocorticoids and their cytosolic receptors have led to the development of selective glucocorticoids receptor agonists (SEGRAs). These agents can selectively transrepress but have no or minimal transactivating properties, thereby achieving anti-inflammatory effect while avoiding many adverse effects. Several potential drug agents are currently under investigation, and preliminary data are encouraging.[37–39]

## Other Mechanisms

Glucocorticoids can act through regulation of mRNA stability. They have been shown to block the production of several pro-inflammatory cytokines by increasing their mRNA degradation rate.[40] In addition, glucocorticoids can repress histone acetylation, leading to repression of inflammatory genes transcription, like GMCSF. At higher doses, they may promote histone H4 acetylation at specific residues, leading to increased genes transcription.[41]

## Non-genomic Action

A more recently recognized and increasingly understood mode of action of glucocorticoids is the *non-genomic action*. This mode of action entails mechanisms that do not directly and initially influence gene expression, and their effects are not blunted by inhibitors of gene transcription such as actinomycin D.[42] Unlike the genomic action, the non-genomic action is characterized by its rapid onset (seconds to minutes) and short duration of action (60-90 minutes). However, similar to the genomic effect, its effect is dose dependent.[43]

Several basic mechanisms of the non-genomic action of glucocorticoids have been identified so far. Different attempts were made to classify these mechanisms. First, the Mannheim classification[44] included two major categories: direct and indirect action. Then came the classification by Haller and colleagues,[45] which included five different categories: effect on membrane lipids, effect on membrane proteins, effect on intracytoplasmic proteins, protein-protein interactions, and interactions with glucocorticoid transporters. Most recently, a classification has been proposed by Stahn and Buttgereit,[16] which has three categories: nonspecific interactions of glucocorticoids with cellular membranes, specific interaction with membrane-bound GRs, and non-genomic effects mediated through binding to the cytosolic GRs. Below, I will focus on the latter classification system and add another category that has been suggested as a non-genomic mode of action of glucocorticoids, which is inhibition of the extraneuronal monoamine transporter–mediated uptake of norepinephrine.

## Inhibition of the Extraneuronal Monoamine Transporter-mediated Uptake of Norepinephrine

Bronchial airway vasculature plays an important role in asthma pathogenesis by contributing to the inflammatory process. Several changes are noted in the vasculature of asthmatic patients’ airways, including vasodilation, hyperperfusion and increased microvascular permeability. These changes are essential to edema formation and inflammatory cell recruitment.[3,46] The airway vasculature is also changed by the chronic inflammation in the airways, leading to new blood vessel formation, a process called *angiogenesis*.[47,48]

The significant increase in airway mucosal blood flow in asthmatic patients with stable asthma or acute exacerbation has been shown in many studies in comparison with healthy subjects.[49,50] Calculated as in the volume of the conducting airways from the trachea to the terminal bronchioles, mean airway blood flow values were 24% to 77% higher in asthmatic patients in comparison with healthy controls.[51,52] The inhalation of fluticasone (880 mcg) or budesonide (400 mcg) significantly decreased blood flow in both groups, but more in asthmatics, with maximal effect about 30 minutes after inhalation and a return to baseline at 90 minutes, a feature that cannot be explained by the genomic effect.[50,53] In addition, vasoconstrictor effect of α_1_ -adrenergic agonists has been shown to be potentiated in the airways of asthmatic patients.[52,54]

**Figure 2:**
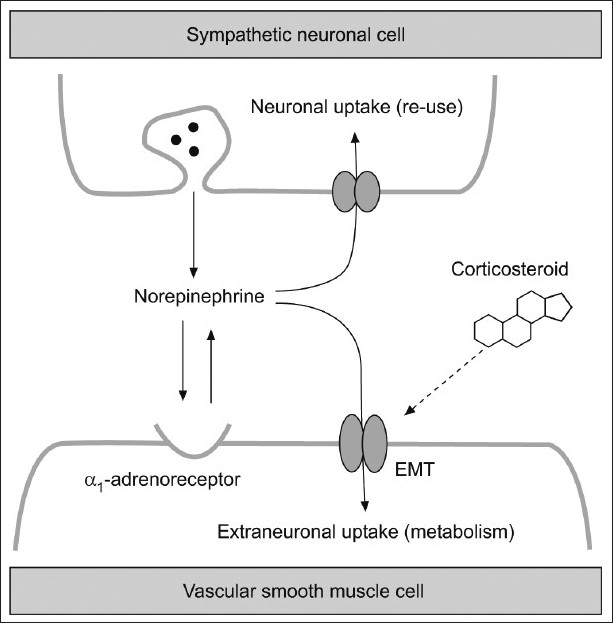
Glucocorticoids facilitate the noradrenergic neuromuscular signal transmission by rapidly (within 5 minutes) inhibiting the extraneuronal monoamine transporter (EMT) in vascular smooth muscle cells. (Adopted from: Horvath, G and Wanner, A. Eur Respir J. 2006;27:172-187 with permission)

How can glucocorticoids cause vasoconstriction? Evidence suggests that glucocorticoids decrease airway blood flow by modulating sympathetic control of vascular tone. Glucocorticoids inhibit the extraneuronal monoamine transporter–mediated uptake of norepinephrine by bronchial arterial smooth muscle cells[55,56] [Figure 2]. Therefore, more epinephrine will be made available in the synaptic cleft to act on the α_1_-adrenoceptors or to be re-uptaken by neurons and released again. Pre-treatment with 5 mg terazosin, a selective α_1_-adrenoceptor antagonist, inhibited the effect of fluticasone on bronchial blood flow, which supports this hypothesis.[43] This effect is a topical, and local one and is a feature of inhaled glucocorticoids rather than systemic glucocorticoids in usual therapeutic doses.[50]

Several lines of evidence from clinical research suggested an important role for this mode of action in the treatment of asthma. A protective effect was demonstrated 2 hours after administration of a single dose of 100 mcg of fluticasone proprionate inhaler using bronchial provocation with cyclic adenosine monophosphate (cAMP).[57] Higher doses were reported to cause more significant effect. In patients with allergic rhinitis, a single large dose of intravenous systemic steroid, that is, 400 mg methylprednisolone or 60 mg betamethasone, resulted in significant decrease in nasal itching within 10 minutes of administration. It took, however, 150 minutes for nasal airflow to be significantly improved.[58] In addition, possibly by diminishing their clearance from the airways, inhaled glucocorticoids were found to potentiate the action of β2 agonists and enhance the onset of action of formoterol, a long-acting β2 agonist.[59,60] On the other hand, inhaled glucocorticoids failed to relieve early airway response represented as changes in FEV1 in some other studies. For instance, a recent study showed that a single dose of budesonide (400 mcg)–formoterol (12 mcg) combination or formoterol alone, but not budesonide alone, given few minutes after airway allergen challenge was able to alleviate early airway response (0-3 hours).[61] Other studies showed similar results regarding the effect of inhaled glucocorticoids.[62,63]

Inhaled glucocorticoids were found to have additional benefit to Systemic glucocorticoids in the management of patients with acute asthma exacerbation in the ER. A recent meta-analysis comparing inhaled to systemic glucocorticoids; or a combination of inhaled and systemic glucocorticoid to a combination of placebo and systemic glucocorticoid in the ER, included 17 different randomized double-blind placebo-controlled trials with data from 1133 subjects (470 adults and 663 children).[64] It concluded that inhaled glucocorticoids present early beneficial effects (within 1 to 2 hours) in terms of clinical and spirometric variables when used in 3 or more doses administered at time intervals of ≤30 minutes over a period of 90 to 120 minutes. Inhaled glucocorticoids led to a significant reduction in admission rate at 2 to 4 hours, with only 10 subjects needing to be treated to prevent one admission.

Taken all together, inhaled glucocorticoids may need to be administered simultaneously with bronchodilators in high and repeated or sequential doses as a way to obtain an early effect and maintain it throughout the time.[65] However, this issue remains controversial, and the current asthma guidelines published by the National Heart, Lung, and Blood Institute (NHLBI)[13] in the USA do not include the use of inhaled glucocorticoids in the treatment of asthma in the ER, while the Global Initiative for Asthma (GINA) guidelines[14] suggested that it can be effective. The author and colleagues are investigating the effect of giving back-to-back doses of nebulized steroid plus β_2_-agonist and ipratropium bromide to children with acute asthma exacerbation, in addition to giving systemic steroids.

Other vascular effects of glucocorticoids have been reported, but their relevance to asthma is not clear.[66–70]

## Interaction with Cellular Membrane

Glucocorticoids can be incorporated into cell membranes, leading to changes in the physicochemical properties of the membrane [Figure 1]. Subsequently, this may interfere with mineral transport across the cell membrane and the cellular production of adenosine triphosphate (ATP).[71,72] These effects result in immune cell suppression. This action is noted at high glucocorticoid doses. In a guinea pig model of asthma, it was shown recently that glucocorticoids inhibit mast cell IgE–mediated exocytosis and histamine release through the reduction of Ca^++^ influx upon induction of allergic inflammation.[73]

## Effects through Membrane-bound Glucocorticoid Receptors

Glucocorticoids were shown to have a rapid T-cell immunosuppressive action that is mediated through membrane-bound GRs [Figure 1]. These receptors were reported to exist in human mononuclear cells and to correlate with glucocorticoids’ lytic responses in lymphoma cells and with disease activity in rheumatoid arthritis.[74,75] These receptors are thought to be variants of cytosolic GRs and not just the classical GRs transported to the cell surface.[74] Liganded receptors inhibit the function of Lck/Fyn kinases, downstream from T-cell receptor, independent from genomic pathways. Inhibition of these enzymes will suppress major pathways important in T-cell activation. This was shown with dexamethasone and prednisolone.[76,77] This mechanism could possibly be of relevance in the treatment of asthma, but an observed clinical effect may take more time than the vasoconstrictor effect.

## Non-genomic Effects through Binding to Cytosolic Glucocorticoid Receptors

Arachidonic acid is produced by the action of cytosolic phospholipase A2 (cPLA2) on membrane phospholipids. By binding to its receptor on the cell membrane, epidermal growth factor (EGF) can activate cPLA2 and therefore arachidonic acid production. Arachidonic acid is a precursor to prostaglandin D_2_ and cysteinyl leukotrienes, which are known to be potent inducers of bronchoconstriction, excessive mucus secretion, and edema of the bronchial mucosa. When glucocorticoids bind their own receptors in the cytosol, some GR protein complex components, like src, dissociate and block the EGF-mediated cPLA2 activation and subsequently arachidonic acid production.[78,79] Although several *in vitro* studies and animal studies have demonstrated decrease in baseline and post-challenge production of cysteinyl leukotrienes after treatment with glucocorticoids, *in vivo* studies generally failed to show a similar effect.[80] This suggests that the action of glucocorticoids through this pathway is not likely to be significant in asthma treatment.

In conclusion, glucocorticoids, as the gold standard asthma therapy, deliver their effects by many ways but most importantly through transrepression by binding key pro-inflammatory transcription factors. Non-genomic mechanisms are being increasingly recognized, especially those affecting bronchial vascular blood flow. Understanding the different mechanisms by which glucocorticoids act should enable the development of new agents that have more desired effects while avoiding many adverse effects by being more specific and selective. It should also open the door for new indications and strategies in the treatment of asthma, things that we may observe in the near future.
